# Stereospecific Inhibitory Effects of CCG-1423 on the Cellular Events Mediated by Myocardin-Related Transcription Factor A

**DOI:** 10.1371/journal.pone.0136242

**Published:** 2015-08-21

**Authors:** Bunta Watanabe, Saki Minami, Hideaki Ishida, Ryuzo Yoshioka, Yoshiaki Nakagawa, Tsuyoshi Morita, Ken’ichiro Hayashi

**Affiliations:** 1 Institute for Chemical Research, Kyoto University, Uji, Kyoto, 611–0011, Japan; 2 Division of Applied Life Sciences, Graduate School of Agriculture, Kyoto University, Kyoto, 606–8502, Japan; 3 Toabo Corporation Co., Ltd., Crystal Tower 18F, 2–27, 1-Chome, Shiromi, Chuo-ku, Osaka, 540–6018, Japan; 4 NAHLS Co., Ltd., Room 2203, Kyodai Katsura Venture Plaza South Building, 1–39 Goryo-Ohara, Nishikyo-ku, Kyoto, 615–8245, Japan; 5 Department of RNA Biology and Neuroscience, Osaka University Graduate School of Medicine, 2–2 Yamadaoka, Suita, Osaka, 565–0871, Japan; Hokkaido University, JAPAN

## Abstract

CCG-1423 suppresses several pathological processes including cancer cell migration, tissue fibrosis, and the development of atherosclerotic lesions. These suppressions are caused by inhibition of myocardin-related transcription factor A (MRTF-A), which is a critical factor for epithelial–mesenchymal transition (EMT). CCG-1423 can therefore be a potent inhibitor for EMT. CCG-1423 and related compounds, CCG-100602 and CCG-203971 possess similar biological activities. Although these compounds are comprised of two stereoisomers, the differences in their biological activities remain to be assessed. To address this issue, we stereoselectively synthesized optically pure isomers of these compounds and validated their biological activities. The *S*-isomer of CCG-1423 rather than the *R*-isomer exhibited modestly but significantly higher inhibitory effects on the cellular events triggered by MRTF-A activation including serum response factor-mediated gene expression and cell migration of fibroblasts and B16F10 melanoma cells. Accordingly, the *S*-isomer of CCG-1423 more potently blocked the serum-induced nuclear import of MRTF-A than the *R*-isomer. No such difference was observed in cells treated with each of two stereoisomers of CCG-100602 or CCG-203971. We previously reported that the N-terminal basic domain (NB), which functions as a nuclear localization signal of MRTF-A, is a binding site for CCG-1423. Consistent with the biological activities of two stereoisomers of CCG-1423, docking simulation demonstrated that the *S*-isomer of CCG-1423 was more likely to bind to NB than the *R*-isomer. This is a first report demonstrating the stereospecific biological activities of CCG-1423.

## Introduction

Epithelial–mesenchymal transition (EMT) is closely linked with cancer cell migration and tissue fibrosis. The activation of myocardin-related transcription factor A (MRTF-A/MAL/MKL1), a transcriptional cofactor for serum response factor (SRF), plays a pivotal role in these pathological processes [1–3]. MRTF-A is also involved in other biological events including development of mammary myoepithelial cells [4, 5], skeletal muscle differentiation [6], and neointimal formation in atherosclerotic lesions [7].

MRTF-A is primarily located in the cytoplasm, but transiently translocates to the nucleus in response to Rho activation. We have recently shown that importin α/β1 heterodimer regulates the nuclear import of MRTF-A in response to Rho activation-induced depletion of G-actin [8]. The N-terminal basic domain (NB) of MRTF-A (B2 [9] or NLS2 [10]), which is positioned between the second and third RPEL motifs, functions as a binding site for importin α/β1 heterodimer. Treisman and co-workers proposed that the importin α/β1 heterodimer interacts with a bipartite nuclear localization signal (NLS) including NB and another N-terminal basic domain (B3) in the second RPEL motif [9], which is also known as NLS1 [10].

CCG-1423, an inhibitor of Rho signaling [11], blocks the nuclear import of MRTF-A. Treatment with CCG-1423 reduces the nuclear accumulation of MRTF-A and improves glucose uptake and tolerance in insulin-resistance mice *in vivo* [12]. This finding suggests that MRTF-A/SRF-mediated pathway is an important target for the development of novel therapeutics for insulin resistance and type 2 diabetes. Furthermore, CCG-1423 inhibits the development of atherosclerotic lesions by reduction in neointimal formation [7]. Thus, these findings suggest that MRTF-A can be an attractive molecular target for drug discovery.

**Fig 1:**
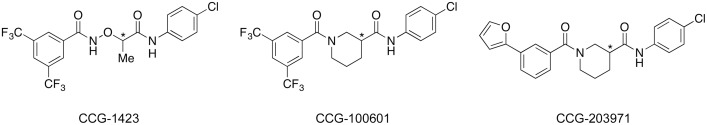
Chemical structures of racemic CCG-1423, CCG-100602, and CCG-203971. The asterisks (*) represent asymmetric centers.

Recently, we have revealed the inhibitory mechanism of CCG-1423 in the nuclear accumulation of MRTF-A. CCG-1423 binds to NB of MRTF-A and inhibits the nuclear import of MRTF-A by masking the NLS [13]. Furthermore, CCG-1423 is suggested to bind other molecules such as MICAL-2, an atypical actin-regulatory protein [14] and Phactr1, a RPEL containing protein [13], indicating the possibility of other mode of CCG-1423. Recent studies reported that CCG-1423 related compounds CCG-100602 and CCG-203971 prevent the nuclear accumulation of MRTF-A in colon and lung fibroblasts [15, 16]. Although each of these compounds is comprised of two stereoisomers, arising from an asymmetric center indicated in Fig 1, the differences in their biological activities remain unclear. It is clinically and pharmaceutically significant to reveal their active structures because the commercially available compounds are a mixture of two stereoisomers. In this study, we stereoselectively synthesized optically pure isomers of CCG-1423 and related compounds (Fig 1), which were developed by Neubig and co-workers. They focused on the inhibitory potential in Rho/ MRTF-A/SRF-mediated pathway [11, 17–19]. We then validated their biological activities and analyzed their binding to MRTF-A by molecular docking simulations. This is a first report demonstrating the stereospecific biological activities of chemical compounds inhibiting the MRTF-A function.

## Materials and Methods

### Chemistry

Optical rotations were measured on a PerkinElmer 341 automatic polarimeter (PerkinElmer, Inc., Waltham, MA, USA). NMR spectra were obtained on JEOL JNM-ECA600 (600 MHz for ^1^H) or JEOL JNM-AL300 (300 MHz for ^1^H) spectrometers (JEOL Ltd, Tokyo, Japan). Chemical shifts are reported in parts per million relative to the internal standards [tetramethylsilane (0.00 ppm) for ^1^H; CD_3_OD (49.00 ppm) for ^13^C]. High performance liquid chromatography (HPLC) was carried out using a Shimadzu LC-10AT VP HPLC system and SPD-10A VP UV detector (Shimadzu Corp., Kyoto, Japan) with CHIRALPAK AD or OD (4.6Φ × 250 mm; Daicel Corporation, Osaka, Japan; detection: UV 254 nm, eluent: *n*-hexane/ethanol, flow rate: 1.0 ml/min, temperature: 30°C).

**Fig 2:**
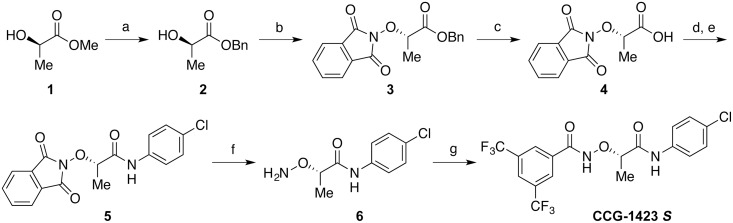
Synthesis of optically active CCG-1423 *S*. Reagents and conditions: (a) benzyl alcohol, iron(III) acetylacetonate, Na_2_CO_3_, heptane, reflux (–H_2_O), 12 h, 87%; (b) *N*-hydroxyphthalimide, dimethyl azodicarboxylate, PPh_3_, THF, RT, 1 h, 63%; (c) H_2_, Pd-C, MeOH, RT, 2 h, 94%; (d) (COCl)_2_, *N*,*N*-dimethylformamide, CH_2_Cl_2_, RT, 1 h; (e) 4-chloroaniline, CH_2_Cl_2_, 0°C, 2 h, 66% (2 steps); (f) NH_2_NH_2_-H_2_O, MeOH, RT, 5 min, 52%; (g) 3,5-bis(trifluoromethyl)benzoic acid, 1-ethyl-3-(3-dimethylaminopropyl)carbodiimide hydrochloride, 4-dimethylaminopyridine, CH_2_Cl_2_, RT, 1 h, 79%.

Thin layer chromatography was carried out using silica gel plates (Merck 5715, 0.25 mm; Merck KGaA, Darmstadt, Germany). Silica gel flash column chromatography was carried out using a Biotage Isolera One chromatograph (Biotage AB, Uppsala, Sweden) with Biotage SNAP Ultra cartridges (silica gel, 25 μm). Methyl (*R*)-lactate and benzyl (*S*)-lactate were purchased from Tokyo Chemical Industry Co., Ltd. (Tokyo, Japan). Both the stereoisomers of 1-(*tert*-butoxycarbonyl)piperidine-3-carboxylic acid were purchased from Alfa Aesar, A Johnson Matthey Company (Heysham, Lancashire, UK). The outline of synthesis of the *S*-isomer of CCG-1423 is summarized in Fig 2.

#### Benzyl (*R*)-lactate (2) [20]

A mixture of methyl (*R*)-lactate (**1**) (5.21 g, 50.0 mmol), benzyl alcolol (5.42 g, 50. 1 mmol), iron(III) acetylacetonate (880 mg, 2.49 mmol), sodium carbonate (270 mg, 2.55 mmol) and heptane (500 ml) was refluxed for 12 h with Dean-Stark apparatus. To the cooled reaction mixture was added silica gen (60 ml) and the solvent was removed under reduced pressure. The residue was subjected to silica gel flash column chromatography (hexane/ethyl acetate = 80/20 ~ 50/50) to afford the title compound (7.83 g, 87%) as a yellow oil. ^1^H NMR (300 MHz, CDCl_3_) δ_H_: 1.43 (3H, d, *J* = 6.8 Hz), 2.80 (1H, d, *J* = 5.3 Hz), 4.32 (1H, qd, *J* = 6.8 and 5.3 Hz), 5.21 (2H, s), 7.29–7.41 (5H, m).

#### Benzyl (*S*)-2-[(phthalimido)oxy]propanoate (3) [21]

To an ice-cooled mixture of compound **2** (3.30 g, 18.3 mmol), *N*-hydroxyphthalimide (3.00 g, 18.4 mmol), triphenylphosphine (4.80 g, 18.3 mmol) in anhydrous tetrahydrofuran (40 ml) was added dimethyl azodicarboxylate (2.0 ml, 18.2 mmol) dropwise and the mixture was stirred for 1 h at room temperature (RT). The reaction mixture was diluted with ethyl acetate followed by addition of silica gel (80 ml). The solvent was removed reduced pressure and the residue was subjected to silica gel flash column chromatography (hexane/ethyl acetate = 85/15 ~ 50/50) to afford the title compound (3.78 g, 63%) as a colorless oil. ^1^H NMR (300 MHz, CDCl_3_) δ_H_: 1.65 (3H, d, *J* = 6.9 Hz), 4.92 (1H, q, *J* = 6.9 Hz), 5.17 (1H, d, *J* = 12.3 Hz), 5.24 (1H, d, *J* = 12.3 Hz), 7.28–7.37 (5H, m), 7.71–7.77 (2H, m), 7.79–7.85 (2H, m).

#### (*S*)-2-[(Phthalimido)oxy]propanoic acid (4) [21]

A mixture of compound **3** (3.77 g, 11.6 mmol) and 5% palladium on activated carbon (410 mg) in methanol (40 ml) was stirred for 2 h under hydrogen atmosphere (1 atm). The catalyst was removed through a pad of Cellite and the filtrate was concentrated under reduced pressure to afford the title compound (2.55 g, 94%) as a pale yellow solid. This was used without purification. ^1^H NMR (300 MHz, CDCl_3_) δ_H_: 1.73 (3H, d, *J* = 6.9 Hz), 4.89 (1H, q, *J* = 6.9 Hz), 7.56 (1H, br s), 7.75–7.89 (4H, m).

#### (*S*)-*N*-(4-Chlorophenyl)-2-[(phthalimido)oxy]propanamide (5)

To an ice-cooled mixture of compound **4** (265 mg, 1.13 mmol) and catalytic amount of *N*,*N*-dimethylformamide in anhydrous dichloromethane (2.5 ml) was added oxalyl chloride (0.20 ml, 2.35 mmol) and the mixture was stirred for 1 h at RT. The reaction mixture was concentrated under reduced pressure to give (*S*)-2-[(phthalimido)oxy]propanoyl chloride as a yellow oil. ^1^H NMR (300 MHz, CDCl_3_) δ_H_: 1.80 (3H, d, *J* = 6.9 Hz), 5.17 (1H, q, *J* = 6.9 Hz), 7.75–7.82 (2H, m), 7.84–7.90 (2H, m). This material was dissolved in anhydrous dichloromethane (2.5 ml) and cooled with the ice-bath. To the solution was added 4-chloroaniline (300 mg, 2.35 mmol) dissolved in anhydrous dichloromethane (5.0 ml) dropwise, and the mixture was stirred for 2 h at 0°C. The reaction mixture was diluted with ethyl acetate (50 ml) and washed successively with 2N HCl, water, and brine (20 ml each). The organic layer was dried over anhydrous magnesium sulfate and concentrated under reduced pressure. The residue was purified by silica gel flash column chromatography (hexane/ethyl acetate = 75/25 ~ 50/50) to afford the title compound (255 mg, 66%) as a pale yellow solid. ^1^H NMR (300 MHz, CDCl_3_) δ_H_: 1.78 (3H, d, *J* = 7.0 Hz), 4.84 (1H, q, *J* = 7.0 Hz), 7.31 (2H, d, *J* = 9.0 Hz), 7.71 (2H, d, *J* = 9.0 Hz), 7.77–7.82 (2H, m), 7.84–7.90 (2H, m), 9.68 (1H, br s).

#### (*S*)-2-Aminooxy-*N*-(4-chlorophenyl)propanamide (6)

To a suspension of compound **5** (215 mg, 0.624 mmol) in methanol (10 ml) was added hydrazine monohydrate (143 mg, 2.86 mmol) and the mixture was stirred for 5 min at RT. The reaction mixture was concentrated under reduced pressure, diluted with water (25 ml), and acidified with 2N HCl (0.5 ml). The insoluble materials were filtered off and the filtrate was washed with ethyl acetate (3 × 20 ml). The aqueous layer was cooled to 0°C, and to the solution was added sodium hydroxide (913 mg) followed by extraction with diethyl ether (3 × 20 ml). The combined organic layer was washed successively with water and brine (15 ml each), dried over anhydrous magnesium sulfate, and concentrated under reduced pressure to afford the title compound (69.8 mg, 52%) as a pale yellow solid. This was used without purification. ^1^H NMR (300 MHz, CDCl_3_) δ_H_: 1.45 (3H, d, *J* = 6.9 Hz), 4.23 (1H, q, *J* = 6.9 Hz), 5.66 (2H, br s), 7.29 (2H, d, *J* = 8.8 Hz), 7.53 (2H, d, *J* = 8.8 Hz), 8.15 (1H, br s).

#### (*S*)-2-{[3,5-Bis(trifluoromethyl)benzoyl]aminooxy}-*N*-(4-chlorophenyl)propanamide (CCG-1423 *S*)

To a mixture of compound **6** (69.8 mg, 0.325 mmol), 3,5-bis(trifluoromethyl)benzoic acid (91.8 mg, 0.356 mmol), 4-dimethylaminopyridie (62.4 mg, 0.511 mmol) in anhydrous dichloromethane (3.5 ml) was added 1-ethyl-3-(3-dimethylaminopropyl)carbodiimide hydrochloride (82.7 mg, 0.431 mmol) and the mixture was stirred for 1 h at RT. The reaction mixture was diluted with ethyl acetate (50 ml), and washed successively with 2N HCl, water saturated aqueous sodium hydrogen carbonate solution, and brine (15 ml each). The organic layer was dried over anhydrous magnesium sulfate and concentrated under reduced pressure. The residue was purified by silica gel flash column chromatography (hexane/ethyl acetate = 70/30 ~ 50/50) to afford the title compound (117 mg, 79%) as a colorless solid. ^1^H NMR (600 MHz, CD_3_OD) δ_H_: 1.60 (3H, d, *J* = 6.8 Hz), 4.66 (1H, q, *J* = 6.8 Hz), 7.31 (2H, d, *J* = 8.6 Hz), 7.68 (2H, d, *J* = 8.6 Hz), 8.17 (1H, br s), 8.39 (2H, s). ^13^C NMR (151 MHz, CD_3_OD) δ_C_: 17.48, 83.79, 122.59 (2C), 124.47 (2C, q, *J*_C-F_ = 272 Hz), 126.34 (br), 128.89 (2C, q, *J*_C-F_ = 4.3 Hz), 129.86 (2C), 130.45, 133.17 (2C, q, *J*_C-F_ = 33.3 Hz), 135.49 (br), 138.02, 172.16, 172.19. The specific rotation ([α]_D_), retention time (rt) and enantiomeric excess (ee) obtained in HPLC analysis, are summarized in Table 1.

**Table 1:** Selected properties of synthetic compounds.

compound	*R*-isomer			*S*-isomer		
	[α]_D_ (°)^a)^	rt (min)^b)^	ee (%)^b)^	[α]_D_ (°)^a)^	rt (min)^b)^	ee (%)^b)^
CCG-1423	+107	43.7^c)^	>99.9	–103	31.2^c)^	>99.9
CCG-100602	–78	8.8^d)^	98.8	+79	5.4^d)^	99.0
CCG-203971	–88	11.3^e)^	99.0	+89	16.7^e)^	99.1

^a)^ Measured in methanol; *c* 1; temperature: 27°C (CCG-1423), 25°C (CCG-100602 and CCG-203971).

^b)^ Determined by HPLC, column: 4.6Φ × 250 mm; eluent: *n*-hexane/ethanol; flow rate: 1 ml/min; temperature: 30°C; detection: UV254 nm.

^c)^ CHIRALPAK AD, *n*-hexane/ethanol = 200/3.

^d)^ CHIRALPAK AD, *n*-hexane/ethanol = 85/15.

^e)^ CHIRALPAK OD, *n*-hexane/ethanol = 85/15.

### Antibodies and plasmids

Antibodies used in this study were as follows: anti-DYKDDDDK (anti-Flag) antibody (Trans Genic, Kobe, Japan), anti-SMα−actin, anti-α−tubulin antibodies, and anti-Flag M2 affinity gel (Sigma, St. Louis, MO), anti-myoD and anti-myogenin antibodies (Santa Cruz Biotechnology, Santa Cruz, CA), anti-myosin heavy chain (MHC) antibody (MF20) (Developmental Studies Hybridoma Bank), and Alexa 568-conjugated secondary antibody (Molecular Probes, Eugene, OR). Concerning a polyclonal anti-MRTF-A antibody, we described in detail previously [13]. Detail information about the plasmids used in this study was described elsewhere [8, 22].

### Cell culture, promoter assay, and immunocytochemistry

Human normal skin fibroblast cell line CCD1059Sk was obtained from the American Type Culture Collection (ATCC). NIH3T3 cells, human skin fibroblasts (ATCC, CRL-2072), and B16F10 melanoma cells were cultured in Dulbecco’s modified Eagle’s medium (DMEM) supplemented with 10% fetal calf serum. Transfection was performed using Trans IT-LT1 (PanVera Corporation, Madison, WI). After transfection, cells were cultured under serum-stimulated conditions for 24 h and were treated with the indicated compounds for further 24 h. Cell lysates were subjected to luciferase and β−galactosidase assays (luciferase assay kit [Promega] and β−galactosidase assay kit [Clonthec]). Relative promoter activity was expressed in luminescence units normalized to the β−galactosidase activity of pSVβ−gal in the cell extracts. These assays were performed in triplicate and were repeated three times. B16F10 cells were stained with anti-MRTF-A antibody and Hoechst 33258. Fluorescent images were analyzed as described previously [23]. The subcellular localization of MRTF-A was divided into three groups: nuclear localization (N); diffuse distribution in the nucleus and throughout the cytoplasm (defined as equivalent immunostaining intensities of the target molecules in the cytoplasm and nucleus) (NC); and cytoplasmic localization (C). In each experiment (n = at least three independent experiments), 100–200 cells were analyzed.

### C2C12 myoblast differentiation

C2C12 myoblasts were usually cultured in DMEM supplemented with 20% fetal calf serum (growth medium [GM]). To induce myogenic differentiation, the culture medium was changed to DMEM supplemented with 2% horse serum (differentiation medium [DM]) at the time point when cells became confluent. Whole cell lysates were prepared at the indicated time point and were subjected to immunoblot (IB) analysis with the indicated antibodies. α−tubulin was used as a loading control. These assays were repeated three times and representative data were shown.

### Wound healing assay

Confluent cells were scratch-wounded with a 20 μl pipette tip. Cell migration was monitored using a low-light inverted Olympus microscope (CKX 41) coupled with a monitoring system, CellPad-E (Tucsen, Fujian, China) every 2 h for 10 h after scratching. Serial 5 images were analyzed with the NIH ImageJ software to quantify the migration area. Percentages indicate the relative migration areas normalized by the migration areas of control cells treated with vehicle, which was set at 100% (means ± SEMs of the results from five serial places). These assays were repeated two and three times.

### Methylthiazole tetrazolium, thiazolyl blue (MTT) assay

Cytotoxicity evaluation of the indicated compounds was performed using The PromoKine Cell Proliferation Assay Kit IV (MTT) (PromoCell, Heidelberg, Germany). Approximately 1 × 10^4^ B16F10 cells were seeded in a 96 well plate and were cultured for 24 h. Cells were treated with the indicated concentrations of each of CCG-1423 and related compounds. After 24 h of incubation, 10 μl of MTT reagent was added to each well and was further incubated for 4 h. Formazan crystals formed in each well were dissolved in DMSO and the plates were read immediately in a microplate reader, SH-9000Lab (CORONA ELECTRIC, Ibaraki, Japan) at 570 nm and 630 nm. These assays were performed in quadruplet and were repeated three times.

### Preparation of cell extracts

The cytoplasmic and nuclear fractions were prepared as described previously [24], and the respective fractions were subjected to IB with the indicated antibodies.

### MRTF-A binding assay using CCG-1423 Sepharose

Preparation of affinity Sepharose covalently coupled with each of the stereoisomers of CCG-1423 was performed based on previously reported methods [13]. MRTF-A protein was synthesized *in vitro* using the TNT SP6 High-Yield Expression System based on an optimized wheat germ extract (Promega) and was purified using anti-Flag M2 affinity gel. Mixtures of MRTF-A protein (300 ng), 0.005% bovine serum albumin, and the indicated CCG-1423 Sepharose or control Sepharose (bed volume 25 μl) in the pull-down (PD) buffer [13] (total 400 μl)] were incubated at 4°C for 2 h with rotation. After washing the respective Sepharose with the PD buffer and phosphate-buffered saline, the pull-downed MRTF-A protein was detected by IB.

### Docking simulation

In order to evaluate the docking precisely, the calculation of the ΔG of the ligand-protein binding is necessary; the ΔG of binding can be calculated using a number of different methods based on molecular dynamics (MD) [25]. However, MD calculations are time consuming. Therefore, scoring functions, where the enthalpy of interaction between the protein and ligand is roughly estimated, were used to speed up the calculations. In this study, the rigid protein and torsionally flexible ligand were employed to calculate a docking score, in which shape and chemical functional complementarity are used. This scoring function is a Gaussian scoring function, which is fundamentally smooth. Gaussian scoring can mimic small fluctuations in protein conformation, allowing modeling of local receptor flexibility. Gaussian-smoothed potentials are applied to measure the complementarity of ligand poses to the active site. In the Chemgauss4 function [26] used in this study, shape interactions, hydrogen bonding interactions with the protein, hydrogen bonding interactions with implicit solvent, and metal-chelator interactions are considered. Comparison of docking simulation between the stereoisomers of each ligand was done using the average of docking scores for the ten highest scored poses for each stereoisomer to reduce the influence of score fluctuation. Prior to running the docking, conformers of each ligand molecule were generated using the conformer generating software, OMEGA (OpenEye, Santa Fe, NM) [27, 28]. Since the conformers of each ligand are generated independent of the active binding site, the running time required for docking experiment can be reduced. In OMEGA, conformers with internal clashes or high strain are discarded, and low strain conformers are clustered on the basis of root mean square deviation. The maximum number of conformers generated by OMEGA is set as 200 in this study.

Crystal structure of the MRTF-A-importin α (PDB: 3TPM) was downloaded from NCBI site (http://www.ncbi.nlm.nih.gov/) and the NLS2 (NB) or NLS1 binding site was excised from the MRTF-A-importin α using MAKE_RECEPTOR tool of OEDocking (OpenEye, Santa Fe, NM). To this excised binding pocket, two stereoisomers of CCG-1423 were docked using Fast Exhaustive Docking of OEDocking (OpenEye, Santa Fe, NM) [26].

### Statistical analysis

Results are given as means and standard errors. Statistical analysis was performed using a two-tailed paired student t-test or a two-way ANOVA when there were multiple data points. The significance level is set at 0.05.

## Results

### Chemistry

The synthesis of chiral CCG-1423 is outlined in Fig 2. Commercially available methyl (*R*)-lactate (**1**) was transesterified by benzyl alcohol in the presence of an iron catalyst [20] to afford benzyl (*R*)-lactate (**2**) in 87% yield. Subsequent Mitsunobu reaction using *N*-hydroxyphthalimide [21] afforded protected hydroxylamine derivative **3** with stereoconversion at the α-positon of the ester carbonyl group. The benzyl ester of compound **3** was removed under catalytic hydrogenation condition to furnish free carboxylic acid **4**. Benzyl ester was suitable in the synthesis since phthalimide group was not stable under strong basic hydrolytic condition.

Next, carboxylic acid **4** was treated with oxalyl chloride in the presence of catalytic amount of *N*,*N*-dimethylformamide, before the resulting acid chloride was reacted with 4-chloroaniline to afford amide **5** in 66% yield. It is noteworthy that standard amidation protocol using carbodiimide coupling reagent such as 1-ethyl-3-(3-dimethylaminopropyl)carbodiimide hydrochloride (EDCl) and base affected *N*-hydroxyphthalimide moiety. In this case, *N*-(4-chlorophenyl)phthalimide was isolated as a major product (37–48% yield) and no desired product was obtained. The phthalimide group of compound **5** was hydrolyzed by hydrazine monohydrate to afford free hydroxylamine **6** in 52% yield. Finally, compound **6** was condensed with 3,5-bis(trifluoromethyl)benzoic acid using EDCl as a coupling reagent and 4-dimethylaminopyridine as a base to furnish desired chiral CCG-1423 (CCG-1423 *S*) in 79% yield. The *R*-isomer of CCG-1423 (CCG-1423 *R*) was synthesized from commercially available benzyl (*S*)-lactate by the same method outlined in Fig 2. The chiral isomers of CCG-100602 and CCG-203971 were synthesized according to the literature method [17, 19] from (*R*) or (*S*)-1-(*tert*-butoxycarbonyl)piperidine-3-carboxylic acid. The selected properties of synthetic chiral compounds were summarized in Table 1. We confirmed optical purity of synthetic compounds by high performance liquid chromatography (HPLC) using chiral columns. All pairs of stereoisomer could be completely separated by CHIRALPAK AD or OD columns, and enantiomeric excess of synthetic compounds was proved to be at least 98.8%.

### Differences in biological activities between racemic and chiral CCG-1423 stereoisomers

**Fig 3:**
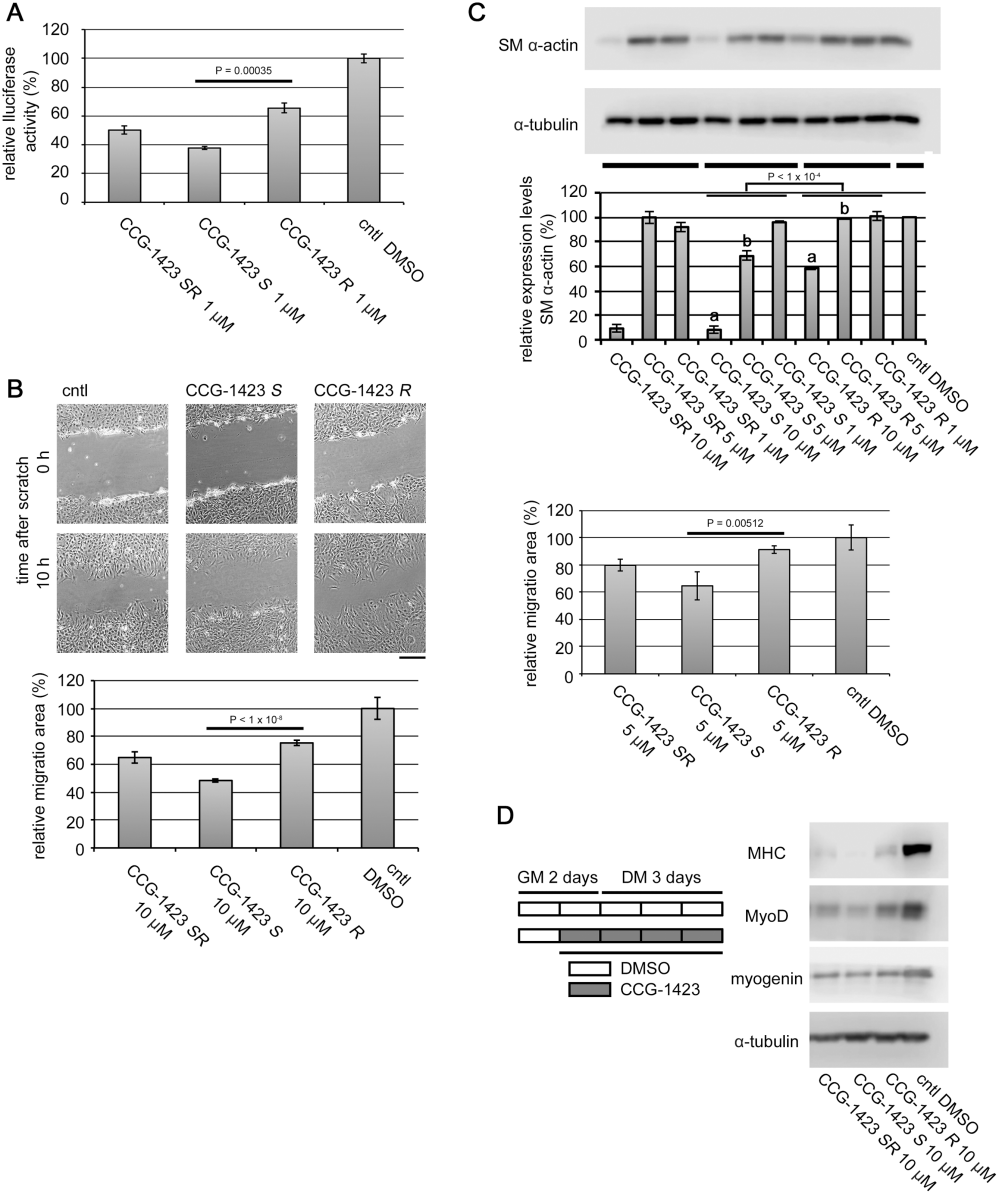
Effects of the racemic and chiral CCG-1423 on the cellular events mediated through MRTF-A/SRF. (A) Promoter analysis using SRF-dependent reporter gene. NIH3T3 cells were transfected with 500 ng of 3xCArG-Luciferase report plasmid, 200 ng of Flag-tagged MRTF-A expression plasmid, and 300 ng of pSVβ−gal. They were cultured for 48 h. For the last 24 h, they were treated with vehicle or 1 μM of racemic or chiral CCG-1423. The luciferase activity with vehicle only (control [cntl] DMSO) was set at 100%. Each value represents the means ± SEMs of results from three independent experiments. Each value represents the means ± SEMs of results from three independent experiments. Statistical differences were calculated using student’s t-test. Significance levels were as follows: CCG-1423 *SR* vs CCG-1423 *S*, P = 0.00240; CCG-1423 *SR* vs CCG-1423 *R*, P = 0.01411; CCG-1423 *S* vs CCG-1423 *R*, P = 0.00035. (B) Wound healing assay. Confluent cultures of NIH3T3 cells were pre-treated with 10 μM of each of the respective compounds or vehicle for 20 h, and then they were scratch-wounded with a 20 μl pipette tip. Bar = 25 μm (upper panel). Cell migration was monitored and quantified as described in Materials and Methods (lower panel). Each value represents the means ± SEMs of results from three independent experiments. Statistical differences were calculated using student’s t-test. Significance levels were as follows: CCG-1423 *SR* vs CCG-1423 *S*, P < 1 × 10^−4^; CCG-1423 *SR* vs CCG-1423 *R*, P = 0.00143; CCG-1423 *S* vs CCG-1423 *R*, P < 1 × 10^−8^. High magnification images were shown in S1 Fig. (C) Effects on the expression of SM α−actin and cell motility of fibroblasts. Human skin fibroblasts were cultured as described in Materials and Methods. For the last 48 h (upper IB analysis) or 20 h (lower cell migration analysis), cells were treated with vehicle or the indicated concentrations of each of the racemic or chiral CCG-1423. Whole cell lysates were subjected to IB analysis with anti-SM α−actin-antibody (upper panels). α−tubulin was used as a loading control. Dose-dependent effects of the respective stereoisomers were analyzed using a two-way ANOVA. The dose-dependent effects between CCG-1423 *S* and CCG-1423 *R* were significantly different (P < 1 × 10^−4^). Significance levels between bar graphs with a and between bar graphs with b were as follows: ^a^P < 1 × 10^−4^ and ^b^P < 1 × 10^−4^. Cell migration was monitored and quantified as described above (lower panel). Each value represents the means ± SEMs of results from three independent experiments. Statistical differences were calculated using student’s t-test. Significance levels were as follows: CCG-1423 *SR* vs CCG-1423 *S*, P = 0.05748; CCG-1423 *SR* vs CCG-1423 *R*, P = 0.00834; CCG-1423 *S* vs CCG-1423 *R*, P = 0.00512. (D) Effect on the differentiation of skeletal myoblasts. C2C12 myoblasts were initially cultured in GM for 2 days, and then were cultured in DM for 3 days. The experimental processes were schematically shown in the left column. White and gray rectangles indicated the cultured period treated with vehicle or 10 μM of racemic or chiral CCG-1423, respectively. Whole cell lysates were prepared at DM 3 day, and they were subjected to IB analysis (right column). α−tubulin was used as a loading control.

Firstly, we compared the effects of the racemic and chiral CCG-1423 on the transcription mediated through MRTF-A/SRF by promoter assay in NIH3T3 fibroblasts. Treatment with racemic (*SR*) or each of the stereoisomers of CCG-1423 [*S*-isomer (*S*) and *R*-isomer (*R*)] significantly reduced SRF reporter activity (Fig 3A). We then examined the effects of the racemic and chiral CCG-1423 on the migration of NIH3T3 fibroblasts. Treatment with the racemic and chiral CCG-1423 suppressed their migration (Fig 3B). In both cases, the most potent reduction in these activities was observed in cells treated with the *S*-isomer of CCG-1423. The hierarchy of the inhibitory effects was *S* > *SR* > *R*. In accordance with these results, similar inhibition was observed in the expression of endogenous SMα−actin protein in human skin fibroblasts (Fig 3C, upper panels). The expression of SMα−actin is also mediated through MRTF-A/SRF pathway. Compared with the inhibitory effect on SRF-mediated transcription by CCG-1423 (1 μM), high doses of CCG-1423 (5 to 10 μM) were necessary for the reduction of SMα−actin expression at the protein levels. Similar hierarchy (*S* > *SR* > *R*) was also observed in the cell migration of human skin fibroblasts (Fig 3C, lower panel). MRTF-A is also involved in myogenesis [29]. Rho signaling-induced activation of MRTF-A in C2C12 myoblasts plays a critical role in their myogenic differentiation [6]. Thus, we examined the effects of the racemic and chiral CCG-1423 on C2C12 myoblast differentiation. Monitoring the expression of myogenic markers suggested that treatment with the racemic and chiral CCG-1423 suppressed differentiation of myoblast into myocyte (Fig 3D). In this case, the *S*-isomer also exhibited higher inhibition activity than the *SR-* and the *R*-isomers: the hierarchy was *S* > *SR* > *R*.

### Differences in biological activities of the stereoisomers of CCG-1423 and related compounds

**Fig 4:**
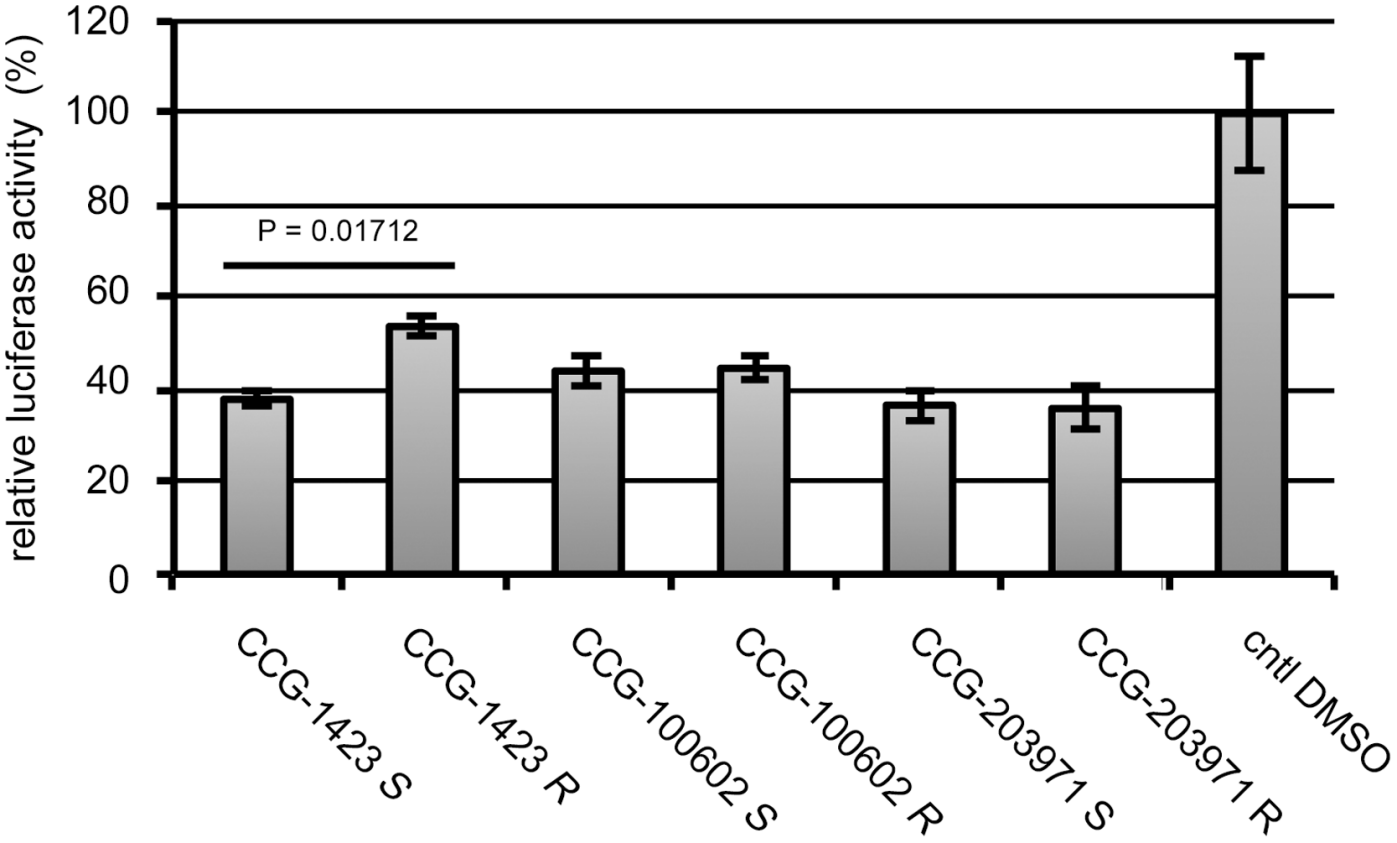
Effects of the stereoisomers of CCG-1423 and related compounds on MRTF-A/SRF-mediated transcriptional activity. Promoter assay in NIH3T3 cells. Detailed procedures were described in Materials and Methods and the legend for Fig 3. For the last 24 h, cells were treated with vehicle or 0.3 μM of the indicated compound. The luciferase activity with vehicle only (cntl DMSO) was set at 100%. Each value represents the means ± SEMs of results from three independent experiments. Statistical differences were calculated using student’s t-test. Significance levels were as follows: CCG-1423 *S* vs CCG-1423 *R*, P = 0.01712; CCG-100602 *S* vs CCG-100602 *R*, P = 0.45219; CCG-203971 *S* vs CCG-203971 *R*, P = 0.45621.

CCG-1423 related compounds, namely CCG-100602 and CCG-203971 (Fig 1), are also comprised of two stereoisomers, but the differences in their biological activities have not yet been characterized. We therefore addressed the effects of the stereoisomers of CCG-1423, CCG-100602, and CCG-203971 on MRTF-A/SRF-mediated transcription by promoter assay (Fig 4). Treatment with the respective stereoisomers of CCG-1423 and related compounds reduced SRF reporter activity. However, among them, significant difference between the *S*- and the *R*-isomers was only observed in cells treated with CCG-1423.

**Fig 5:**
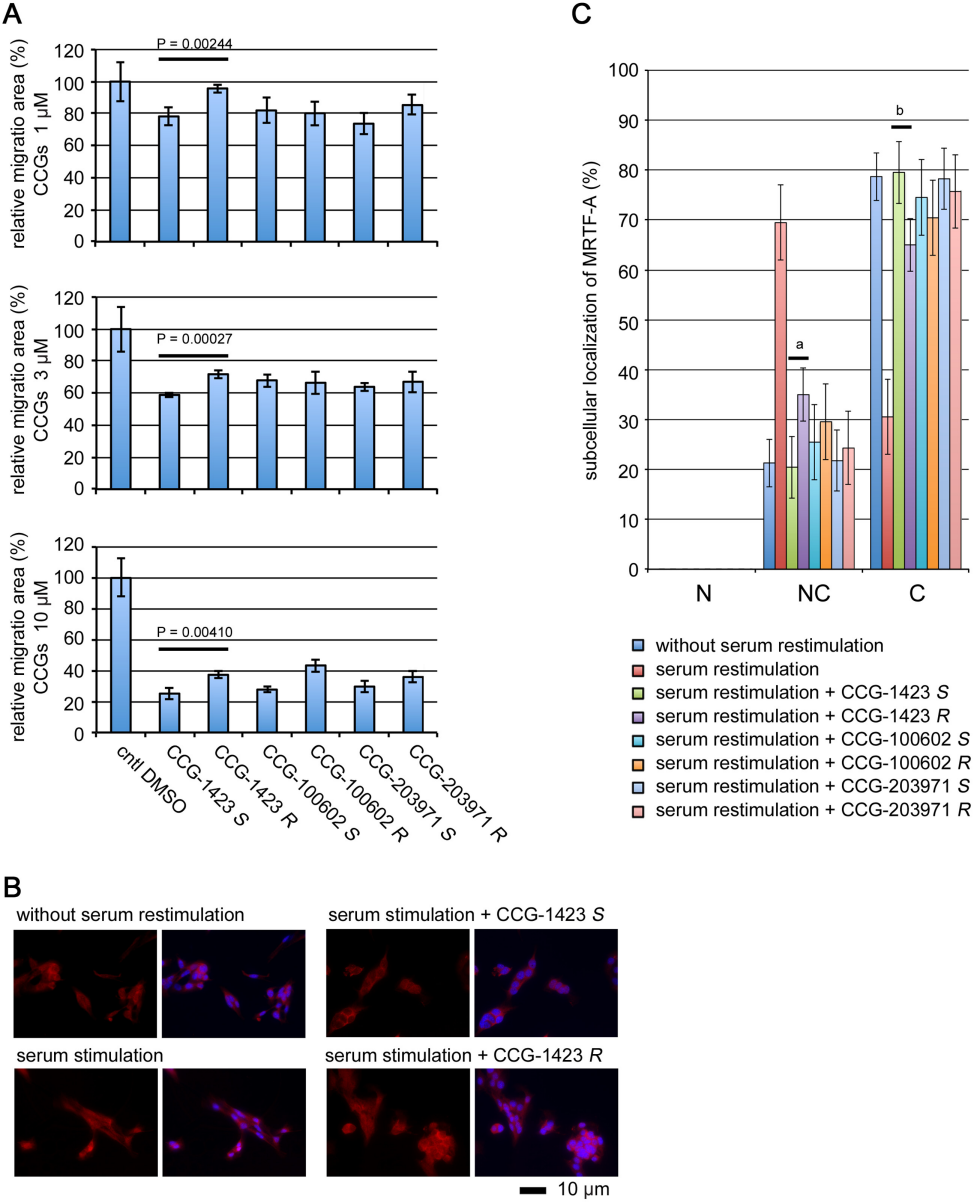
Effects of the stereoisomers of CCG-1423 and related compounds on cell migration and nuclear import of MRTF-A. (A) Confluent cultures of B16F10 cells were pre-treated with the indicated concentrations of each of the respective compound or vehicle for 12 h, and then they were scratch-wounded with a 20 μl pipette tip. Cell migration was monitored and quantified as described in Materials and Methods. Statistical differences were calculated using student’s t-test. (B and C) B16F10 cells cultured in DMEM-10% serum were pre-treated with 3 μM of the indicated compound or vehicle for 12 h, and then they were restimulated with serum for 15 min (final serum concentration 20%). Vehicle-treated B16F10 cells with or without serum restimulation were used as controls. Cells were stained with anti-MRTF-A antibody (red) and Hoechst (blue). Representative images of control cells and CCG-1423 treated cells are shown (B). The images were analyzed as described in Materials and Methods: nuclear-specific localization [N], diffuse distribution in the nucleus and the cytoplasm [NC], and cytoplasmic localization [C] (C). Each value represents the means ± SEMs of results from three independent experiments. Subcellular localization of MRTF-A was statistically analyzed by a two-way ANOVA. The dose-dependent effects between CCG-1423 *S* and CCG-1423 *R* were significantly different (P < 1 × 10^−4^). Significance levels were as follows: ^a^P < 1 × 10^−4^ and ^b^P < 1 × 10^−4^.

Activation of MRTF-A/SRF pathway plays a critical role in the migration but not in the proliferation of B16 melanoma cells [30]. We therefore examined the effects of the stereoisomers of CCG-1423 and related compounds on the migration of B16F10 cells (Fig 5A). Treatment with these stereoisomers reduced cell migration in a dose-dependent manner. At any concentration tested in this study, the *S*-isomer of CCG-1423 exhibited higher inhibitory potency than the *R*-isomer of CCG-1423. However, no such difference was observed in cells treated with either CCG-100602 or CCG-203971. We also analyzed the subcellular localization of endogenous MRTF-A in B16F10 cells treated with each of these stereoisomers (Fig 5B and 5C, S2 Fig). Serum-induced nuclear import of MRTF-A was modest in B16F10 cells; MRTF-A was diffusely localized both in the nucleus and throughout the cytoplasm but was not localized only in the nucleus in most of cells restimulated with serum. Treatment with these stereoisomers markedly increased the proportion of cells exhibiting the cytoplasmic localization of MRTF-A, suggesting that the nuclear import of MRTF-A is inhibited. The inhibitory effect of the *S*-isomer of CCG-1423 was higher than that of the *R*-isomer. We also examined the cytotoxic effects of these compounds by MTT assay (S3 Fig). Every compound reduced cell viability in a dose-dependent manner. Compared with CCG-1423, the cytotoxic effects of CCG-100602 and CCG-203971 were modest. Furthermore, the cytotoxic effect of the *S*-isomer of CCG-1423 was slightly but significantly lower than that of the *R*-isomer of CCG-1423. However, such difference was not observed in the cytotoxic effects between the stereoisomers of CCG-100602 and CCG-203971.

### Computer-associated binding simulation of the stereoisomers of CCG-1423

**Fig 6:**
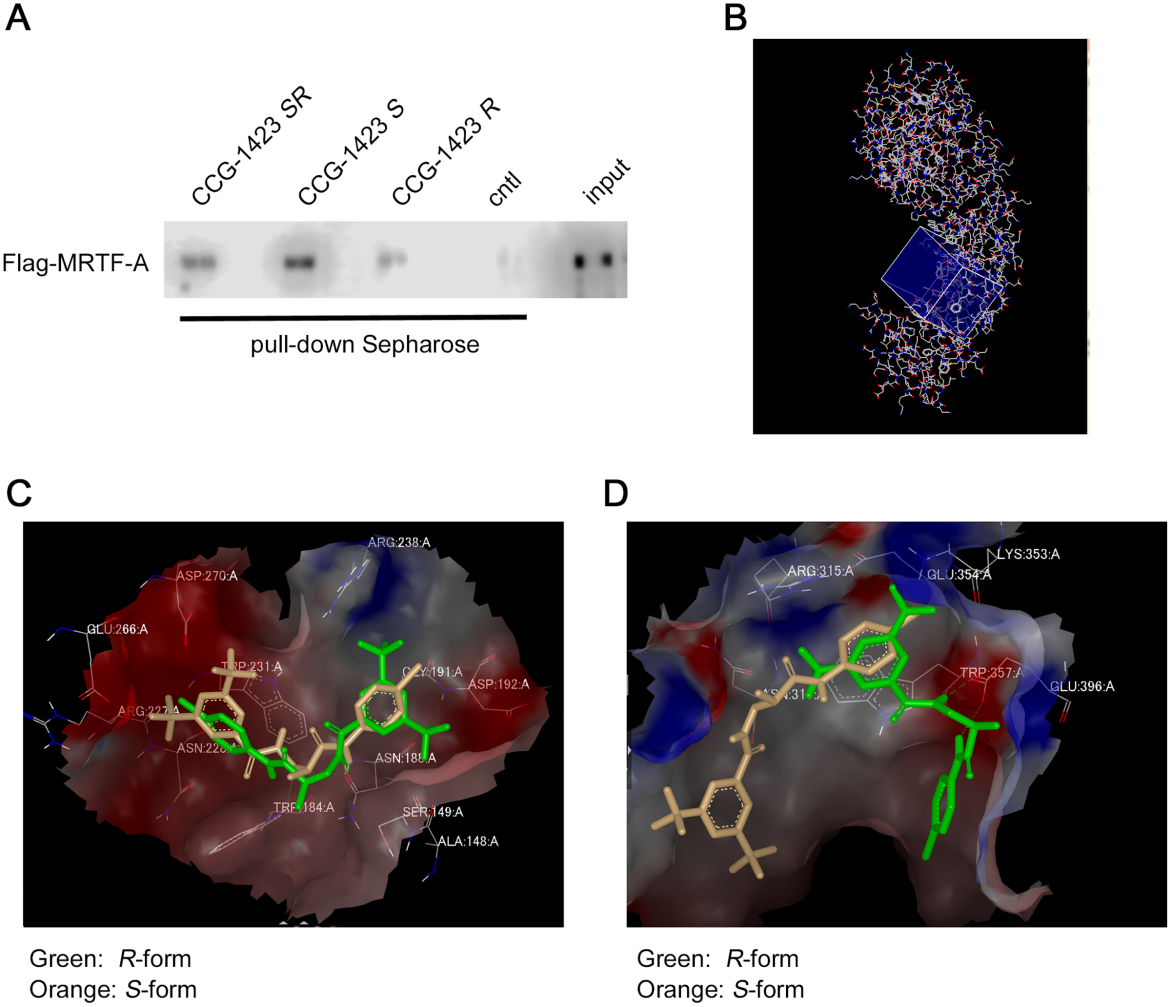
Docking simulation of the stereoisomers of CCG-1423. (A) *In vitro* binding of MRTF-A to each of the racemic or chiral CCG-1423. Purified *in vitro* translated Flag-MRTF-A protein was incubated with Sepharose coupled with each of the racemic or chiral CCG-1423 or control Sepharose (cntl). Detailed procedures are described in Materials and Methods. Flag-MRTF-A protein bound to the respective Sepharose was detected by IB with anti-DYKDDDDK antibody. (B) The squared blue box is chosen for the docking simulation, which is constructed by MAKE_RECEPTOR. The volume size of this squared box is 5594Å^3^. (C) The NB docking site is excised from the crystal structure (3TPM). Chemical structures shown with orange color is the *S*-isomer of CCG-1423, and green is for the *R*-isomer. Both isomers form hydrogen bonds between NH close to 3,5-dimethylbenzoyl moiety of CCG1423 and -CO- group of side chain moiety of asparagine (ASN188). The hydrogen bond distances for the *S-* and *R*-isomers are 1.77Å and 2.02Å, respectively. Red and blue spheres are electrostatically negative and blue fields for the receptor. (D) Docking simulation of the *S*-isomer (orange) and the *R*-isomer (green) of CCG1423 to NLS1 binding site. Red and blue fields are electrostatically negative and positive regions of the binding pocket.

In previous study, we found that racemic CCG-1423 directly binds to the NLS of MRTF-A (NB) and inhibits the binding of importin α/β1 heterodimer [13]. We therefore compared the binding affinities of the respective stereoisomers of CCG-1423 for MRTF-A using Sepharose coupled with racemic or chiral CCG-1423. The binding affinity for MRTF-A was decreased in the following order: *S* > *SR* > *R* (Fig 6A). This finding well coincided with their biological activities shown in Fig 3.

Crystal structure of MRTF-A RPEL domain (residues 67–186) is reported by Hirano and Matsuura [10], in which two NLSs were unambiguously identified: NLS1 (^118^LKRK^121^) and NLS2 (^151^LKLKRARLAD^160^). NLS2 is corresponded to NB, and the sequence of NB (KLKRAR) within NLS2 is identified as a functional NLS of MRTF-A (8). We therefore performed the docking simulation focused on NB binding pocket. The volume size of the trimmed protein (boxed by grid in Fig 6B) including NB biding pocket is 5594 Å^3^ (19.00 Å × 16.67 Å × 17.67 Å). Two stereoisomers were docked to this binding pocket. The *S*-isomer of CCG-1423 showed higher docking score (-4.30785 ± 0.16283 for the *S*-isomer and -3.43768 ± 0.27834 for the *R*-isomer, P < 0.05), but the interactions based on hydrogen bond between respective isomers and NB site were similar. Their docking patterns to NB binding pocket are shown in Fig 6C. Both the stereoisomers docked to NB binding pocket in a similar manner, but their binding direction was opposition of left and right. These findings suggest that the *S*-isomer of CCG-1423 exhibits a higher affinity for NB binding pocket than the *R*-isomer, but drastic difference in their binding affinities is not found. On the other hand, Treisman and co-workers proposed that importin α/β1 heterodimer interacts with a bipartite NLS including NB (B2 or NLS2) plus another N-terminal basic domain (B3 or NLS1) in the second RPEL motif [9]. In order to get another supportive evidence for the docking sites for CCG-1423, we performed the docking simulation focusing on this domain. The docking models to the NLS1 binding pocket were largely different between the *S*- and *R*-isomers (Fig 6D). Since the biological activities were not dramatically different between two stereoisomers (Fig 3), the NLS1 binding pocket is not plausible as the target site for CCG-1423.

## Discussion

MRTF-A is functionally involved in several pathological conditions including cancer cell migration and tissue fibrosis [29]. MRTF-A therefore can be an attractive molecular target for drug discovery and many researchers have paid attention to the screening of MRTF-A inhibitor. In this study, we stereoselectively synthesized optically pure isomers of CCG-1423 and related compounds and validated their biological activities. As a result, we found the stereospecific effect of CCG-1423 on the cellular events activated by MRTF-A. Although Kim *et al*. have recently reported the difference in the anti-EMT activity between the stereoisomers of ginsenoside 20-Rg3 [31], this is a first report demonstrating the stereospecific biological activities of chemical compounds inhibiting the MRTF-A function. These present findings provide useful information for the drug discovery focusing on the inhibition of MRTF-A function.

In this study, we successfully synthesized the target compounds from chiral starting materials. The values of specific rotation ([α]_D_) of each pair of the stereoisomers strongly suggested that the respective pairs are in mirror image relationship because only the +/–signs are different (Table 1). The optical purity of synthetic compounds was clearly confirmed by chiral HPLC analysis, and the enantiomeric excess of synthetic compounds was proved to be at least 98.8%. Based on these observations, no apparent racemization occurred during the aforementioned synthetic process.

Among the compounds that we tested, CCG-1423 only exhibits the stereoisomer-specific differences in the inhibitory effects on MRTF-A/SRF-mediated cellular functions. The *S*-isomer of CCG-1423 rather than the *R*-isomer potently suppressed the gene expression mediated by SRF activation, cell migration, and nuclear import of MRTF-A (Figs 3, 4 and 5). However, CCG-100602 and CCG-203791 did not exhibit such stereoisomer-specific differences. In accordance with the finding that the binding affinity of the *S*-isomer CCG-1423 for MRTF-A was higher that that of the *R*-isomer (Fig 6A), molecular docking simulation revealed that the *S*-isomer of CCG-1423 shows modestly more stable binding to NB site, which is identified as a target site of CCG-1423 in MRTF-A molecule (13). To enhance the binding affinity of the inhibitors for MRTF-A, introducing of a covalent bond-forming functional group such as *N*-hydroxysuccinimidyl or *p*-toluenesulfonyl esters into the inhibitors might be promising because NB of MRTF-A is rich in nucleophilic lysine residue. Complex formation between the inhibitors and NB would strongly inhibit the binding of importin α/β1 heterodimer to MRTF-A. Two benzene ring of CCG-1423 and related compounds would be suitable site for introducing a covalent bond-forming functional group because our docking simulation suggests that around the sites of NB seem to be able to accommodate bulky substituents. Of interest, the *R*-isomer of CCG-1423 had a greater cytotoxic effect than the *S*-isomer of CCG-1423 (S3 Fig). These results suggest that CCG-1423 differently affect the MRTF-A-mediated cellular events and cell viability. In contrast, CCG-100602 and CCG-203971 did not show such features.

In conclusion, our novel findings are as follows: (1) the *S*-isomer of CCG-1423, rather than the *R*-isomer, shows higher potency in the inhibition of MRTF-A/SRF-mediated gene expression, cell migration, and serum-induced nuclear import of MRTF-A, (2) Molecular docking simulation reveals that the *S*-isomer of CCG-1423 exhibits modestly higher binding affinity for the NLS of MRTF-A than the *R*-isomer. These findings provide a valuable information resource for drug design to block epithelial–mesenchymal transition.

## Supporting Information

S1 Fig High magnification images of Fig 3B. Bar = 25 μm.
(TIF)

S2 Fig Analysis of subcellular localization of MRTF-A in B16F10 cells by immunoblot (IB). B16F10 cells cultured in DMEM-10% serum were pre-treated with 3 μM of the indicated compound or vehicle for 12 h, and then they were restimulated with serum for 15 min (final serum concentration 20%). Their cytoplasmic (C) and nuclear (N) fractions were subjected to IB with the indicated antibodies. Vehicle-treated B16F10 cells with or without serum restimulation were used as controls. α−tubulin and histone H2B were used as loading controls for the cytoplasmic and nuclear fractions, respectively. Representative results from two independent experiments are shown.
(TIF)

S3 Fig Effects of the stereoisomers of CCG-1423 and related compounds on the viability of B16F10 cells. Viabilities of B16F10 cells treated with the indicated compounds were assayed by MTT assay as described in Materials and Methods. The viability with vehicle only (cntl DMSO) was set at 100%. Each value represents the means ± SEMs of results from three independent experiments. Dose-dependent effects of the respective stereoisomers were analyzed using a two-way ANOVA. The dose-dependent effects between CCG-1423 *S* and CCG-1423 *R* were significantly different (P < 1 × 10^−4^). Significance level between bar garphs with a was P = 0.0001.
(TIF)
