# Supplementary figures and images for: Stereospecific Inhibitory Effects of CCG-1423 on the Cellular Events Mediated by Myocardin-Related Transcription Factor A
Source: PLoS One. 2015 Aug 21;10(8):e0136242. doi: 10.1371/journal.pone.0136242 (PMC4546662; doi:10.1371/journal.pone.0136242)

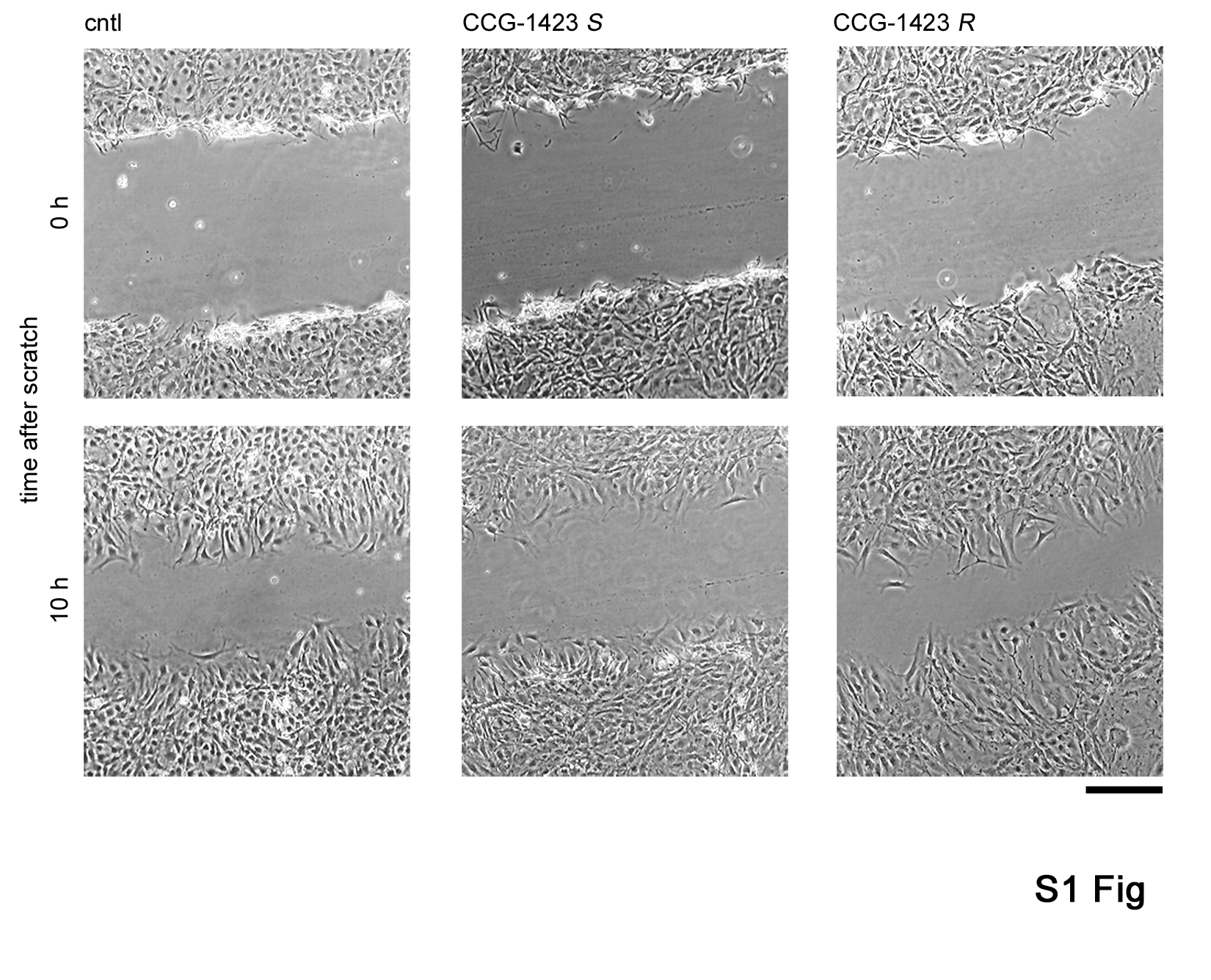

Supplement: S1 Fig — Bar = 25 μm. (TIF) [file pone.0136242.s001.tif]

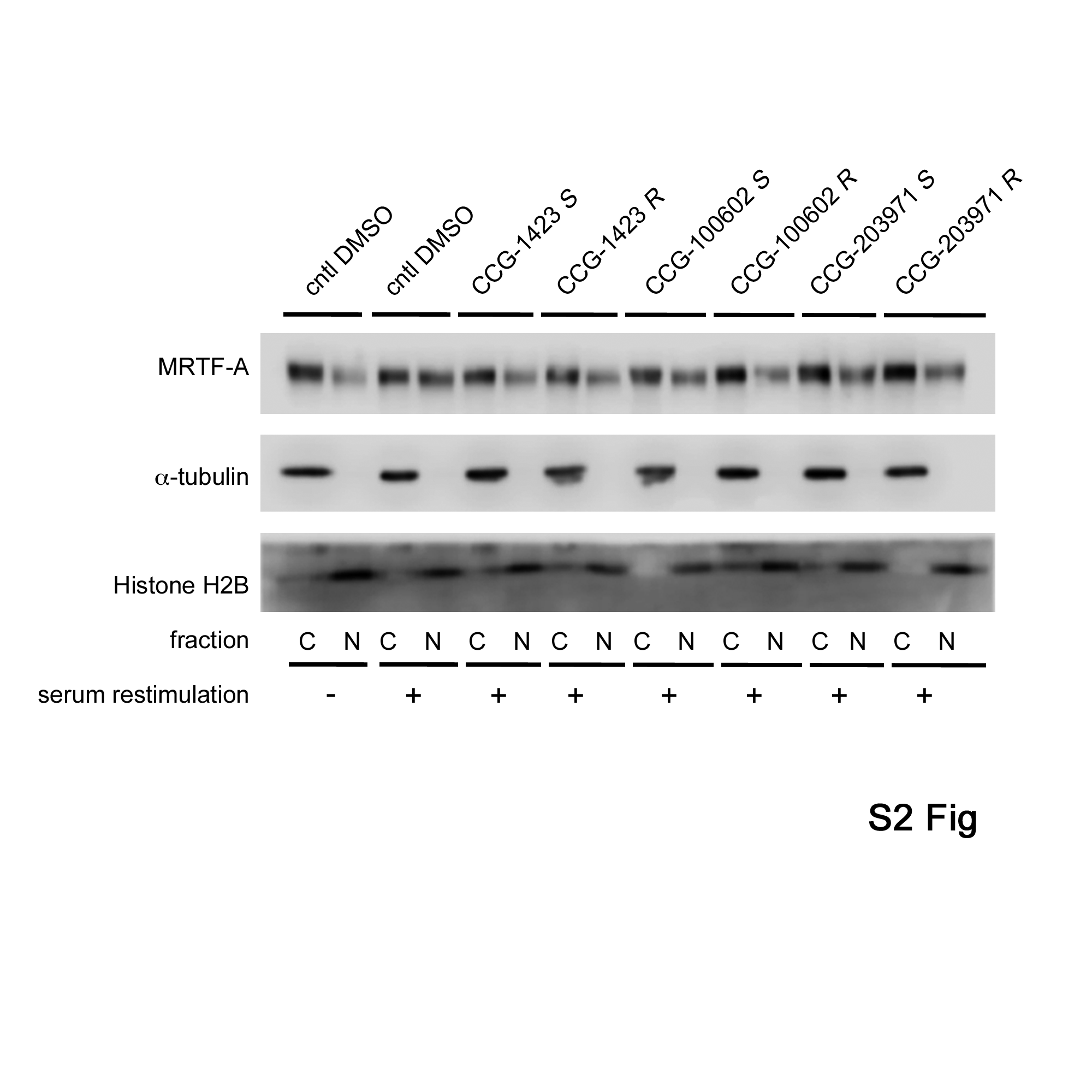

Supplement: S2 Fig — B16F10 cells cultured in DMEM-10% serum were pre-treated with 3 μM of the indicated compound or vehicle for 12 h, and then they were restimulated with serum for 15 min (final serum concentration 20%). Their cytoplasmic (C) and nuclear (N) fractions were subjected to IB with the indicated antibodies. Vehicle-treated B16F10 cells with or without serum restimulation were used as controls. α−tubulin and histone H2B were used as loading controls for the cytoplasmic and nuclear fractions, respectively. Representative results from two independent experiments are shown. (TIF) [file pone.0136242.s002.tif]

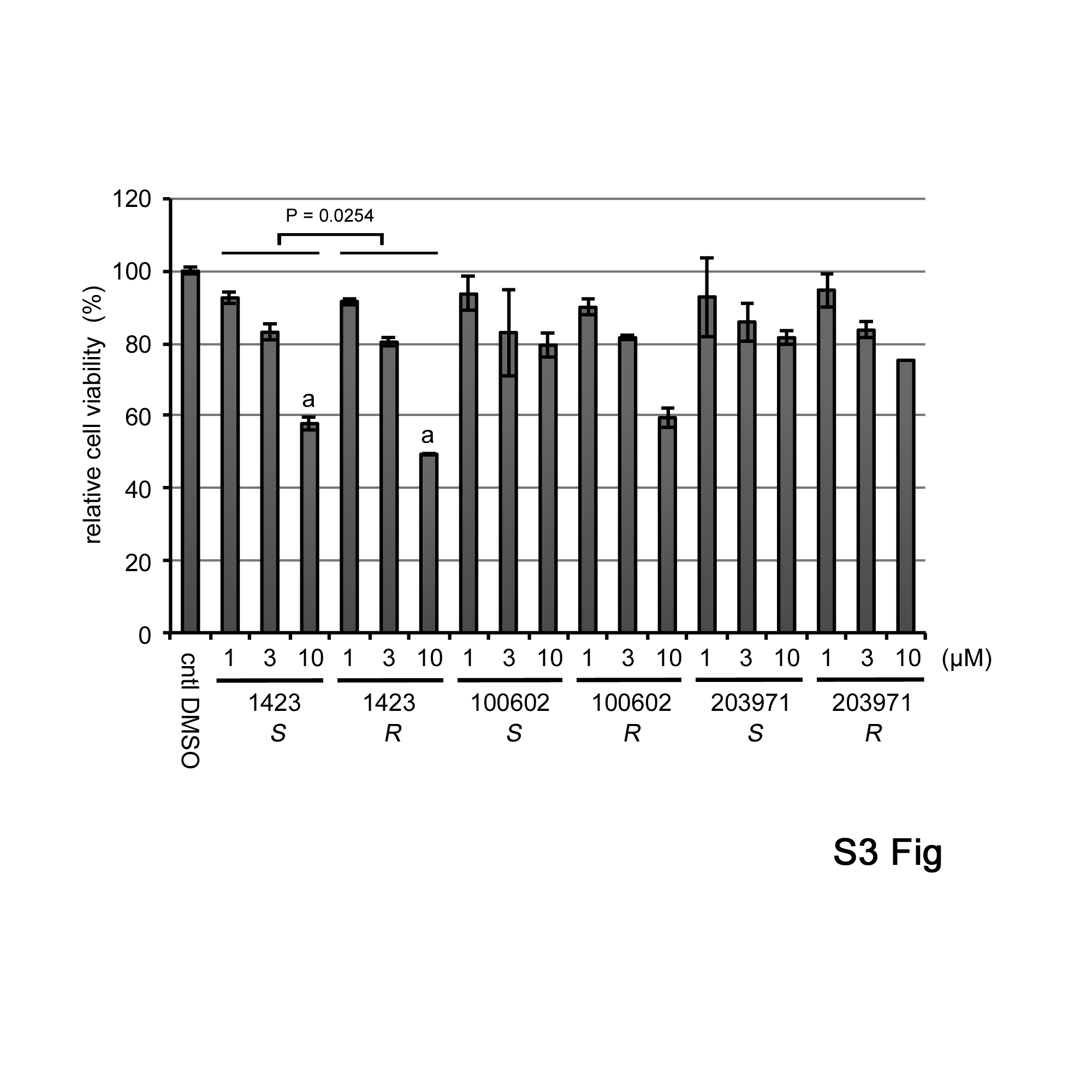

Supplement: S3 Fig — Viabilities of B16F10 cells treated with the indicated compounds were assayed by MTT assay as described in Materials and Methods. The viability with vehicle only (cntl DMSO) was set at 100%. Each value represents the means ± SEMs of results from three independent experiments. Dose-dependent effects of the respective stereoisomers were analyzed using a two-way ANOVA. The dose-dependent effects between CCG-1423 S and CCG-1423 R were significantly different (P < 1 × 10^−4^). Significance level between bar garphs with a was P = 0.0001. (TIF) [file pone.0136242.s003.tif]
